# Genome-Wide Analysis of Artificial Mutations Induced by Ethyl Methanesulfonate in the Eggplant (*Solanum melongena* L.)

**DOI:** 10.3390/genes10080595

**Published:** 2019-08-07

**Authors:** Xi-ou Xiao, Wenqiu Lin, Ke Li, Xuefeng Feng, Hui Jin, Huafeng Zou

**Affiliations:** 1 South Subtropical Crop Research Institute Chinese Academy of Tropical Agricultural Sciences, Zhanjiang City 524091, China; 2 Zhanjiang City Key Laboratory for Tropical Crops Genetic Improvement, Guangdong Province, Zhanjiang City 524091, China

**Keywords:** eggplant, ethyl methanesulfonate mutation, fruit color, gene function, whole-genome re-sequencing

## Abstract

Whole-genome sequences of four EMS (ethyl methanesulfonate)-induced eggplant mutants were analyzed to identify genome-wide mutations. In total, 173.01 GB of paired-end reads were obtained for four EMS-induced mutants and (WT) wild type and 1,076,010 SNPs (single nucleotide polymorphisms) and 183,421 indels were identified. The most common mutation type was C/G to T/A transitions followed by A/T to G/C transitions. The mean densities were one SNP per 1.3 to 2.6 Mb. The effect of mutations on gene function was annotated and only 7.2% were determined to be deleterious. KEGG (Kyoto Encyclopedia of Genes and Genomes) pathway analysis showed 10 and 11 genes, which were nonsynonymous mutation or frameshift deletion in 48-5 and L6-5 involved in the anthocyanin biosynthesis or flavone and flavonol biosynthesis. QRT-PCR results showed that only the Sme2.5_06210.1_g00004.1, which was annotated as UFGT (Flavonoid galactosidase transferase), expression significantly decreased in the L6-5 mutant compared with the WT. Also, the Sme2.5_06210.1_g00004.1 expression was lower in the colorless eggplant compared with colorful eggplant in the natural eggplant cultivar. These results suggest that Sme2.5_06210.1_g00004.1 may play a key role in eggplant anthocyanin synthesis.

## 1. Introduction

Eggplant (*Solanum melongena* L.) is an important vegetable in sub-tropic and tropic areas. According to the FAO (Food and Agriculture Organization) database, eggplant production is 52,309,119 tons worldwide in 2017 (FAO). Eggplants are not only used as food but also in medicine to benefit human health [1]. Although eggplants exhibit diverse phenotypes regarding fruit shape, color, and taste, geneticdiversity is narrow [2]. Therefore, it is of utmost importance to develope eggplant germplasms for the purposes of eggplant breeding and molecular studies.

EMS (ethyl methanesulfonate) is a common agent for inducing mutations and has been widely used in plants, such as tomatoes [3,4], peppers [5,6], eggplants [7], and *Arabidopsis* [8]. EMS-induced mutations include single nucleotide polymorphism (SNPs), base transition, base transversion and insertions, and deletions (indels). The mechanism of the EMS-induced mutation is that EMS induces mutations through the alkylation of guanines, which causes thymine to mis-pair with O-6-ethyl G instead of cytosine. Genome-wide research has shown that C/G to T/A transitions are predominant mutations in EMS-induced mutants [9,10,11,12]. Intron and intergenic mutations are also predominant mutations [12]. EMS-induced mutants exhibit a variety of phenotypes, metabolic products, and biotic/abiotic stress tolerance [3,4,7,13,14]. Genetic mutants not only contribute to breeding programs but also possess great potential to further molecular studies [13].

Based on phenotypic and genotypic variations, mutants are screened by forward and reverse genetic methods. For forward genetic methods, based on the mutation character, the segregation of the mutant and wild type is conducted and then the mutations can be mapped and cloned by a map-based cloning approach, such as leaf rust disease resistance gene, *Lr10* [15]. Recently, high-throughput sequencing has been applied as a forward genetic method that identifies candidate genes within a few months [16]. New methods, such as MutMap [13] and MutMap+ [17], have been developed to clone mutations in EMS-induced mutants. For reverse genetic methods, gene function information is annotated, and genes are identified by methods, such as high-resolution melting or single-strand conformation polymorphism. The most popular technology for reverse genetics is Targeting Induced Local Lesions IN Genomics, which enables researchers to screen mutant libraries on a large scale with low cost [8,18]. Besides the Targeting Induced Local Lesions IN Genomics approach, genome-wide analysis of the EMS-induced mutations is also a reverse-genetic approach for mutant screening [9,12]. Genome-wide re-sequencing results showed that there are abounds of SNPs and indels between the WT and EMS-induced mutants, which include spontaneous mutations and EMS-induced mutations [9,12]. However, to our best knowledge, the approach to filter spontaneous mutations and confirm the key EMS mutation, which affects the phenotype, is limited. In common, the spontaneous mutations were considered as common homozygous mutations and then those mutations were filtered [12,19]. The remaining mutations were considered as EMS-induced mutations and were analyzed further [9,12]. Analysis of the effects of the EMS-induced mutations on gene function identified the high-impact mutations (nonsense mutations and frameshift mutations) that may help to clone the key EMS-mutation, which affects the phenotype. KEGG pathway mapping and GO (Gene Ontology) annotation analysis are efficient methods to analyze gene function [20]. Analysis of the deleterious mutations’ gene functions by KEGG pathway mapping and GO annotation is feasible.

In our prevision study, eggplant seeds treated with EMS exhibited diversity in physiology and metabolites in M_2_ generation [7]. In this study, whole-genome re-sequencing was performed on wild type and four EMS-induced mutants to characterize the mutations. 

## 2. Materials and Methods

### 2.1. Plant Material

The EMS-induced mutants were obtained in our previous study (Xi-ou et al., 2017) [7]. Four EMS-induced mutants of the M_2_ generation eggplants were selected and transplanted in the field located at the South Subtropical Crop Research Institute Chinese Academy of Tropical Agricultural Sciences (21°10′2″ N; 110°16′34″ E). Phenotypes are described in Table 1. After 25 days self-spollination, 6 eggplant fruit were harvested and the fruit diameter and fruit length were measured.

### 2.2. Illumina Sequencing Analysis

One plant of each mutants’ line was randomly selected, and the genomic DNA was extracted from leaves using a DNeasy Plant Mini Kit (Qiagen, Hilden, Germany), as per the manufacturer’s instructions. Genomic DNA purity was analyzed using a NanoDrop^®^ spectrophotometer (Thermo Fisher, Waltham, MA, USA), and DNA concentration was measured using the Qubit^®^ DNA Assay Kit in a Qubit^®^ 3.0 Fluorometer (Life Technologies, Carlsbad, CA, USA). Approximately 1 μg of quality genomic DNA was used for sequencing library construction. The sequencing libraries were generated using the VAHTS Universal DNA Library Prep Kit for Illumina^®^ (Vazyme, Najing, China), as per the manufacturer’s instructions. The sequencing library was sequenced on an Illumina Hiseq X Ten platform using a 150 bp paired-end module.

### 2.3. SNPs’/Indels’ Identification and Annotation

After removing low-quality reads (reads containing adapter; the reads containing ploy-N and the number of base, which is Q ≤ 10 is more than 50% of the entire read) clean reads were mapped to the eggplant reference genome sequence (Sme2.5) [19] using Burrows-Wheeler Aligner software with default parameters. Alignment files were converted to BAM files using SAMtools software. SNPs and indels were identified using Genome Analysis Tool Kit software [20]. The annotation and effects of mutations on gene function were predicted using ANNOVAR software [21].

### 2.4. GO and KEGG Pathway

The high-impact mutation genes, which were nonsense mutations and frameshift mutations, were analyzed by GO and the KEGG pathway. The analysis first maps all high-impact mutation genes to the biological process, cellular component, and molecular function terms in the Gene Ontology database (http://www.geneontology.org/). This calculates the number of genes for each term, and then applies a hypergeometric test to find out the genotype. The GO entries were significantly enriched in a high-impact mutations gene. Pathway significance enrichment analysis using KEGG pathway as a unit applies hypergeometric tests to find pathways that are significantly enriched in high-impact mutations gene compared to the entire genomic context.

### 2.5. Expression Analysis

The eggplant peel was collected at 15 days after self-pollination and then immediately frozen in the liquid nitrogen and stored at −80 °C. Three eggplants peel was mixed as a repeations and there were 3times repeations The total RNA of the eggplant peels of L6-5 and 48-5 was extracted as the column plant RNAout 2.0 kit manual (Tian Enze Beijing). Next, 1 µg RNA was synthesized into cDNA with Oligo dT18 as per the manufacturer’s instruction (Takara Dalian). Gene expression was analyzed by a Roche LightCycler 480 thermal cycler. In total, 10 µL reaction mix contain 5 µL 2X Maxima SYBR Green qPCR Master Mix (Thermo fisher), 0.8 µL primers, 1 μL cDNA, and 3.2 µL RNase-free water. The amplification program was as following: 95 °C for 3 min; 95 °C for 15 s, 60 °C for 30 s, 72 °C for 15 s, 45 cycles. The primers used in this study are listed in Table S1.

### 2.6. Sanger Sequencing

The DNA of the WT and L6-5 were used as template for cloning the Sme2.5_06210.1_g00004.1 full length. The primer was Sme2.5_06210.1_g00004.1F ATGAATGGGAACTCAAATG: and Sme2.5_06210.1_g00004.1R: CTCATGGTTATTTGAGAGCTTAGC. In total 25 μL the reaction mix contain 12.5 μL PrimeSTAR® Max DNA Polymerase(takara ), 1 μL Sme2.5_06210.1_g00004.1F, 1 μL Sme2.5_06210.1_g00004.1R, 1μL DNA and 9.5 μL sterile water. The amplification program was as following: 95 °C for 35 min; 95 °C for 30 s, 52 °C for 30 s, 72 °C for 30 s, 30 cycles and 72 °C for 5 min. The the PCR products was sequenced by Tian Yi Hui Yuan.

## 3. Results

### 3.1. Whole-Genome Re-Sequencing of Five Eggplant Lines

To identify the EMS-induced mutations, we obtained a total of 173.01 GB of paired-end reads for five eggplant lines, including a wild-type line and four EMS-induced mutants (Table 2). For the four EMS-induced mutants, the average depth was approximately 30× and the coverage was approximately 98% (Table 2). These results suggest that the sequence was suitable for SNP and indels analysis.

### 3.2. Identification of Single Nucleotide Substitutions and Indels

**Figure 1:**
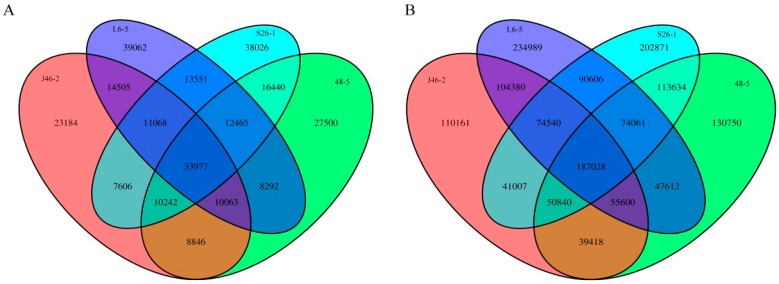
A Venn diagram showing the numbers of SNPs (Single Nucleotide Polymorphisms) that overlapped between the four EMS (ethyl methanesulfonate)-induced mutants. (**A**) SNPs between the four EMS-induced mutants, (**B**) indels between the four EMS-induced mutants.

After reads were mapped to the eggplant genome reference sequence Sm 2.5, candidate mutations were filtered using the following criteria: 1) Quality scores of >50; 2) read depths between 10, 100, and 3) genotyping scores of ≥20 [12]. In total, 1,832,327 candidate mutations were obtained, including 1,557,500 SNPs and 274,827 indels (<12 bp). SNPs and indels common among three random mutant lines were filtered to remove spontaneous occurrences (Figure 1). Among the four mutants, 187,028 SNPs and 33,977 indels were common. After filtering, 1,076,010 SNPs and 183,421 indels were identified. Of the SNPs, 678,771 were unique and 397,239 were common between two mutants. Of the indels, 127,772 were unique and 55,649 were common between two mutants. Among the four EMS-induced mutants, the L6-5 mutants showed the highest number of unique SNPs (477587) and indels (75273) followed by the S26-1 mutants (Table 3).

### 3.3. Characterization of the SNPs and Indels

**Figure 2:**
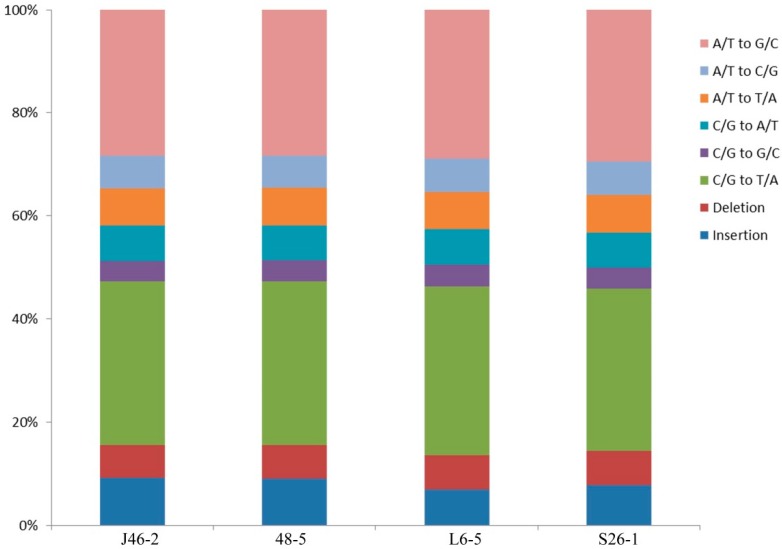
Genome wide analysis the proportions of SNPs mutations in the four ethyl methanesulfonate-induced eggplant mutants.

The mean densities were 1 SNP/2.6 Mb, 1 SNP/2.3 Mb, 1 SNP/1.3 Mb, and 1 SNP/1.7 Mb in the J46-2, 48-5, L6-5, and S26-1, respectively (Table 3). The maximum density was 568.5 Mb in the S26-1 mutant. In all four mutants, the minimum SNPs density was 1 bp. SNPs comprised 581,042 C/G to T/A transitions (37.4%); 523,964 A/T to G/C transitions (33.7%); 116,478 A/T to C/G transversions (7.5%); 132,149 A/T to T/A transversions (8.5%); 124, 343 C/G to A/T transversions (8.0%); and 74,109 C/G to G/C transversions (4.8%) (Figure 2). The ratio of transitions to transversions was 6.52. In the four mutants, the C/G to T/A transitions was the most frequent mutation and the A/T to G/C transitions was the second most common.

**Figure 3:**
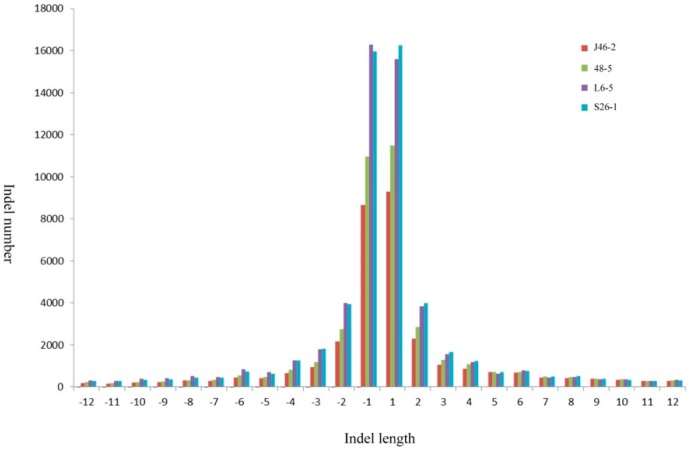
Genome-wide analyses the proportions of indels mutations in the four ethyl methanesulfonate -induced eggplant mutants.

The mean densities were 1 indel/14.7Mb, 1 indel/13.0 Mb, 1 indel/10.5 Mb, and 1 indel/10.5 Mb in the J46-2, 48-5, L6-5, and S26-1, respectively. The maximum density was 550.3 Mb in the L6-5. The minimum density was 1 bp for all four mutants (Table 3). The most frequent indels observed were a 1 bp insertion (52,626) and 1 bp deletion (51,843) (Figure 3). The largest insertion and deletion were 12 bp.

### 3.4. Effects of Mutations on Gene Function

The functional effects of the SNPs and indels were predicted and classified into three impact categories based on mutation type: High-impact (nonsense mutations and frameshift mutations), moderate-impact (intron and intergenic mutations), and low-impact (synonymous mutations). Of the four mutants, L6-5 (28,489) had the most SNPs with high-impact mutations (27,220) followed by S26-1 (Table 4).

High-impact mutations cause a loss of gene function, thereby becoming the focus of further analysis. There were 5318, 5071, 9045, and 5636 genes in the J46-2, 48-5, L6-5, and S26-1 that were high-impact mutations.

### 3.5. Gene Ontology (GO) Annotation

**Figure 4:**
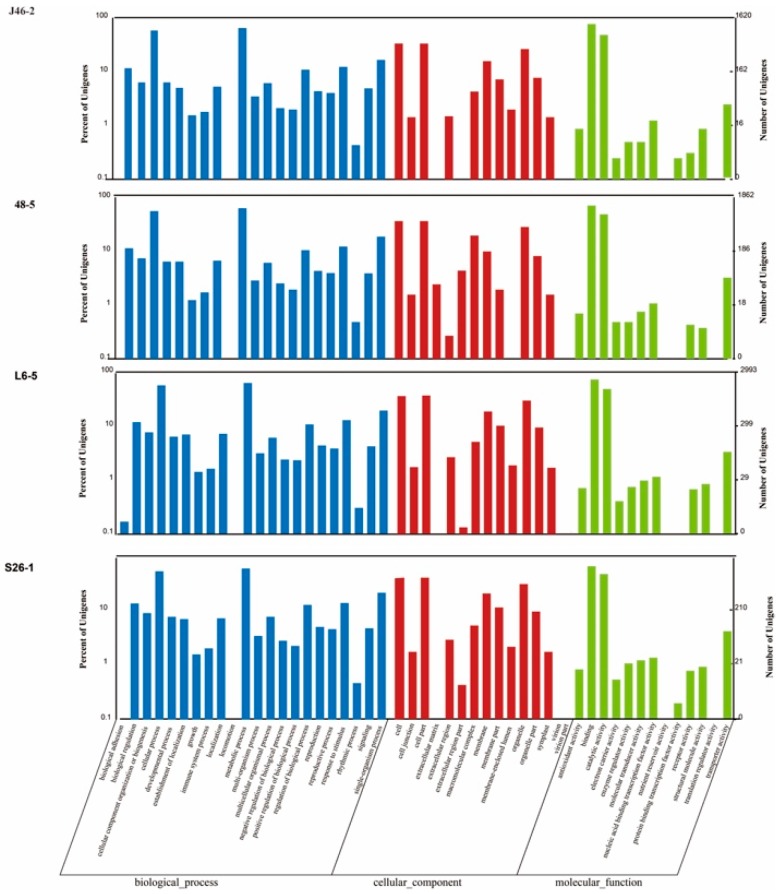
GO classification of the high-impact mutations gene in the four ethyl methanesulfonate -induced eggplant mutants. The high impact mutation genes, which include nonsense mutations and frameshift mutations genes, were assigned to three main categories: biological process, cellular components, and molecular function.

There were 1620, 1862, 2993, and 2701 genes, which were high-impact mutations assigned GO terms in the J46-2, 48-5, L6-5, and S26-1 (Figure 4). For the biological process category, “cellular process” and “metabolic processes” were the most frequently assigned GO terms in the four EMS mutants. For the cellular component category, “cell”, “cell part”, and “organelle” were the most frequently assigned GO terms in the four EMS-induced mutants. For the molecular function category, “binding” and “catalytic activity” were the most frequently assigned GO terms in the four EMS-induced mutants.

### 3.6. KEGG Pathway Mapping

There were 2889, 3108, 4950, and 3327 genes, which were high-impact mutations in the J46-2, 48-5, L6-5, and S26-1 mutants that were mapped to the KEGG pathway. Those genes were predicated 102 pathways, 120 pathways, 120 pathways, and 118 pathways in J46-2, 48-5, L6-5, and S26-1, respectively. The most represented pathways were homologous recombination (Pathway ID: ko03440), RNA degradation (Pathway ID: ko03018 ), ribosome biogenesis in eukaryotes (Pathway ID: ko03008), metabolic pathways (Pathway ID:ko01100), and biosynthesis of secondary metabolites (Pathway ID: ko01110) in all four mutant (Table S2).

### 3.7. The Expression of Sme2.5_06210.1_g00004.1 Decreased Significantly in the L6-5 Mutant

Among the four mutants, 48-5 and L6-5 exhibited a white fruit color. This phenotype indicated a loss-of-function or nonsynonymous mutation of the gene regulating anthocyanin synthesis. *MYB1*, *bHLH*, *PAL*, *CHI*, *F3H*, *F3’5’H*, *DFR*, *ANS*, *AN11*, *CHS*, and *3GT* (Table S3) are involved in eggplant anthocyanin synthesis [7,21,22]. However, no SNPs or indels were found in these genes in either the 48-5 or L6-5 mutants.

**Figure 5:**
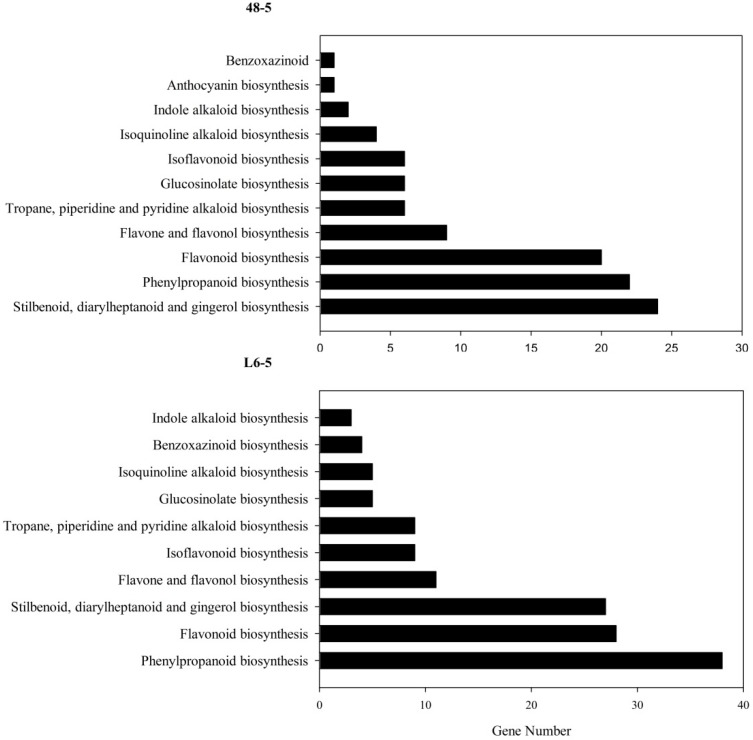
Biosynthesis of other secondary metabolites KEGG of the high-impact mutations gene in the four EMS-induced eggplant mutants. The high-impact mutations gene include the nonsense mutations and frameshift mutations genes. 48-5 and L6-5 were white fruit mutants.

**Figure 6:**
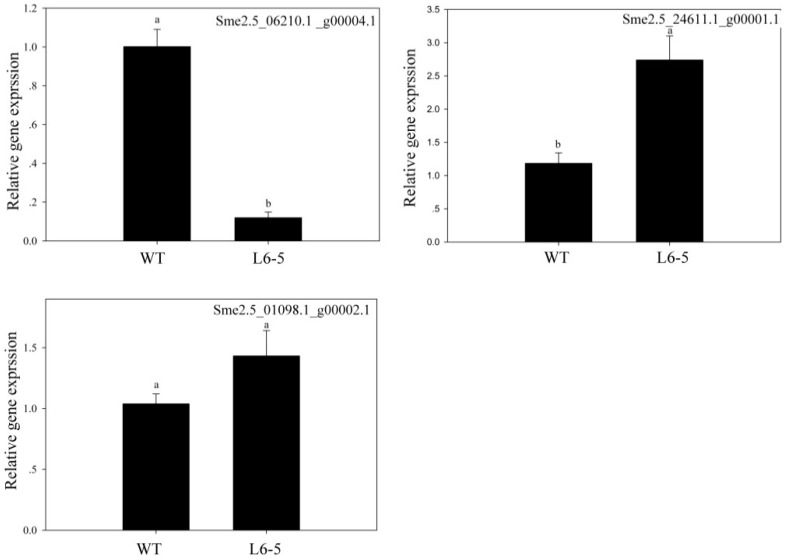
The gene expression analysis of nonsense mutations genes which involved in the flavone and flavonol biosynthesis pathway. in the L6-5. The L6-5 was the white fruit mutants and the nonsense mutations genes were mapped to the flavone and flavonol biosynthesis.

**Figure 7:**
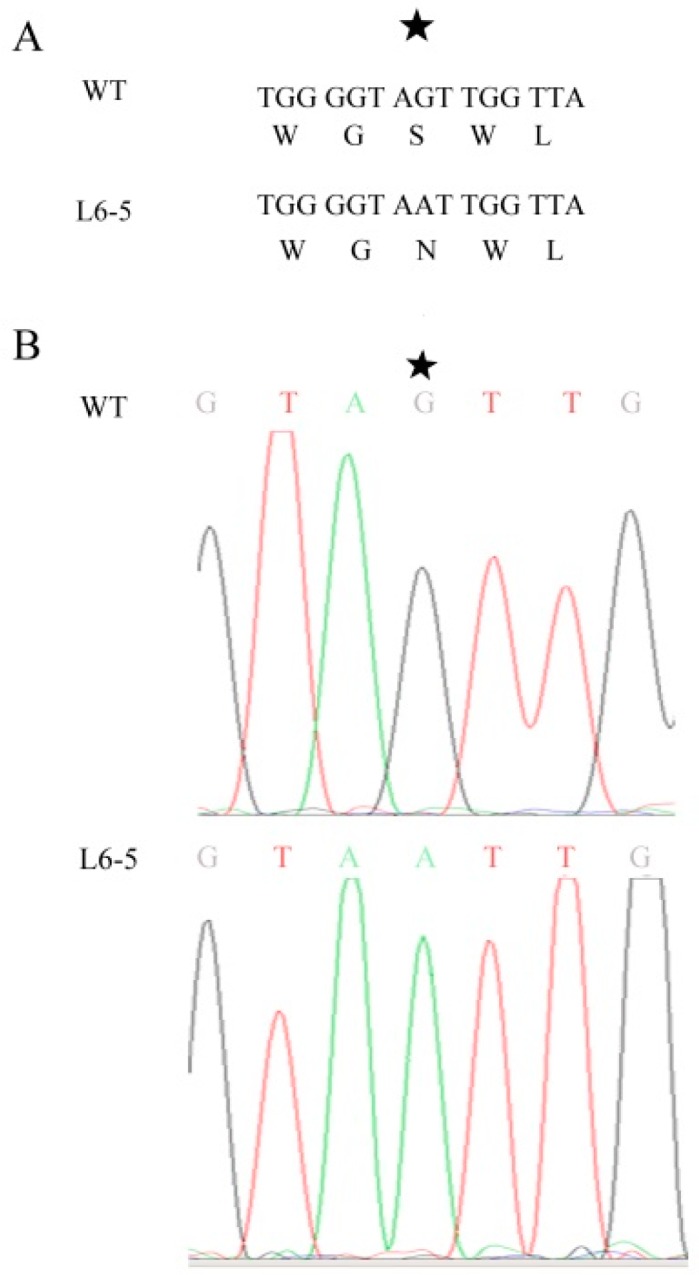
The SNP of Sme2.5_06210.1_g00004 between the wild type and L6-5. (**A**) The SNP of Sme2.5_06210.1_g00004.1 at the 785bp (G–A), which results in the amino acid Ser mutant to Asn, (**B**) Sanger sequencing identified the SNP. The asterisk indicates the SNP.

Because the anthocyanin was a type of flavone and flavonol, the gene involved in the flavone and flavonol biosynthesis pathway (Pathway ID: ko00944) and anthocyanin biosynthesis pathway (Pathway ID: ko00942) were analyzed. The result showed that in the 48-5 mutants, one anthocyanin biosynthesis gene (Sme2.5_03583.1_g00011.1), and nine flavone and flavonol biosynthesis genes were mapped (Figure 5, Table 5). However, in the L6-5 mutant, 11 flavone and flavone genes were mapped (Figure 5, Table 5). To further analyze the mutant gene function in the eggplant anthocyanin synthesis, those genes expression level were detected by RT-PCR. However, Sme2.5_00384.1_g00003.1, Sme2.5_00928.1_g00014.1, Sme2.5_01087.1_g00007.1, Sme2.5_03930.1_g00006.1, Sme2.5_05662.1_g00003.1, Sme2.5_13126.1_g00003.1, Sme2.5_1399 6.1_g00001.1, Sme2.5_24902.1_g00001.1, Sme2.5_26073.1_g00001.1, and Sme2.5_03583.1_g00011.1 expression were undetectable in both 48-5 mutant and WT. In the L6-5 mutant, the Sme2.5_00081.1_g00002.1, Sme2.5_01191.1_g00004.1, Sme2.5_02030.1_g00005.1, Sme2.5_02274.1_g00006.1, Sme2.5_07919.1_g00001.1, Sme2.5_12449.1_g00001.1, Sme2.5_1312 6.1_g00003.1, Sme2.5_00081.1_g00002.1, and Sme2.5_26073.1_g00001.1 was undetectable. Only Sme2.5_06210.1_g00004.1, Sme2.5_01098.1_g00002.1, and Sme2.5_24611.1_g0000 1.1 expression were detected in L6-5 and WT. The result showed that the Sme2.5_01098.1_g00002.1 expression was increased in the L6-5. However, only Sme2.5_06210.1_g00004.1 expression significantly decreased in the L6-5 compared with the WT (Figure 6). Am SNP of Sme2.5_06210.1_g00004.1 at 785 bp (G-A_) was the result of the amino acid Ser mutant to Asn. Also, the SNP were identified by the Sanger sequencing (Figure 7).

## 4. Discussion

In the present study, we analyzed EMS-induced SNPs and indels in eggplants by whole-genome re-sequencing. Results revealed that the four EMS-induced mutants contain abundant SNPs and indels as compared to the WT eggplant. The effects of the SNPs and indels on gene function were also analyzed.

Genome re-sequencing is the most effective approach to identify genetic diversity induced by chemical and physical mutagenesis and is also an effective approach for cloning target genes. Several million SNPs and indels have been reported in mutants [9,11,12,23]. When analyzing mutants, it is important to filter for spontaneous SNPs and indels. There are two strategies to filter spontaneous SNPs and indels in EMS-induced mutants: Analyzing a large genome sequence database and removing common SNPs [12,24] or re-sequencing pooled F_2_ segregation population DNA and identifying target SNPs with the SNP-index [13,17,23,25]. An effective approach is to filter the common SNPs and indels between different plants. Shirasawa et al. [12] analyzed SNPs in seven wild-type Micro-Tom lines and considered 1,211,647 common SNPs as spontaneous. After filtering, only 5920 of the common SNPs were considered causal SNPs in EMS-induced mutants. Uchida et al. [9] re-sequenced the genome of the F_2_ generation of EMS-induced mutants (Ws background) crossed with Col-T. After the common SNPs between the F_2_ generation Ws (32,142) and Col-T (34,757) were conducted, only 24 and 34 were considered causal, respectively. In our present study 1,557,500 SNPs and 274,827 indels were detected before filtering. Due to the available genome sequence data of the eggplant, only the SNPs and indels common between three random mutants were considered spontaneous. After filtration, only 481,490 SNPs and 91,406 indels were removed. Eventually 1,076,010 SNPs and 183,421 indels were obtained, which proved to be too many to determine which caused the phenotype change.

The proposed mechanism of EMS-induced mutagenesis is that guanines are alkylated and then paired with thymine. Adenines then replace guanines during DNA replication (Greene et al. 2003). Therefore, C/G to T/A transitions (>99%) are the most common mutation type [24] and are targeted when analyzing mutations linked to changes in the phenotype [25]. In the present study, C/G to T/A transitions (37.4%) were the most frequent, consistent with the previous results in EMS-induced tomatoes (35.6%–73.3%) [12] and rice (80%) [23]. Other transitions have been reported in tomatoes [12], cucumbers [25], and soybeans [10]. Alkylation of nitrogen can occur with G at N-7 or A at N-3, forming 3-ethyladenine, which results in G/C to C/G or T/A transversions and A/T to G/C transitions, respectively [18,26].

Genome sequencing was used to identify mutations that led to a change in phenotype. The effects of the mutations on gene function were analyzed with a focus on high-impact mutations. Our results suggest that the whole-genome NGS technique is a convenient approach for identifying genes associated with phenotypic variation with high-impact mutations. In the present study, we found 28,489 SNPs with high-impact effects on 24,247 genes. However, a lack of genome sequence information impacted the efficiency of filtering for spontaneous SNPs, hindering identification of the genes associated with phenotypic variation in EMS-induced eggplants.

Eggplant fruit color is determined by anthocyanin, which is delphinidin-3-glucoside-5-(coumaryl) dirhamnoside [21,22]. The biosynthetic pathway of the anthocyanin has been well-characterized [21], and the genes involved in anthocyanin biosynthesis in eggplants have been analyzed [27]. In the present study, 11 genes reported by Zhang et al. [21] in the anthocyanin biosynthesis pathway did not contain SNPs or indels in the L6-5 and 48-5, which had white fruit, indicating disruption of anthocyanin biosynthesis. These results suggest that novel gene mutations lead to the change in fruit color observed in the L6-5and 48-5 mutants. Then, the KEGG pathway mapping showed that in the 48-5 mutants, one anthocyanin biosynthesis gene and 11 flavone and flavonol biosynthesis gene were mapped. In the L6 mutant, 11 flavone and flavonol genes were mapped.

In the L6-5 mutants, only expression of Sme2.5_06210.1_g00004.1 were significantly decreased compared with the WT. The result is consistent with the eggplant peel transcriptome analysis between L6-5 and WT (date not published). Sme2.5_06210.1_g00004.1 was an annotation as anthocyanidin 3-O-glucosyltransferase (3GT) in the NCBI database. 3GT played a key role in plant anthocyanidin synthesis, which catalyzes the transfer of glucose from UDP-glucose to anthocyanidins, such as delphinidin [28]. In Japanese apricot, an SNP mutation leading to nonsynonymous mutations affects the petals’ variegation [29]. Also, Li et al. [22,30] showed that Sme2.5_06210.1_g00004.1 expression was up-regulated during eggplant anthocyanidin synthesis. However, Zhang et al. [21] and Li et al. [22] showed that other 3GT (Sme2.5_00228.1_g00013.1) also may play an important role in eggplant anthocyanidin synthesis. The nucleic acid and protein sequence alignment between Sme2.5_06210.1_g00004.1 and Sme2.5_00228.1_g00013.1 is shown in Figure S1. The result indicated that Sme2.5_06210.1_g00004.1 is a novel 3GT gene. The expression and annotation analysis suggested that Sme2.5_06210.1_g00004.1 may play a key role in eggplant anthocyanidin synthesis (Figure S2). However, the function of the Sme2.5_06210.1_g00004.1 in eggplant anthocyanidin synthesis will be further analyzed by knocked down in the WT and overexpression in the L6-5.

To map or clone mutations causing fruit color change, WT fruit was hybridized with L6-5 and 48-5 mutants. The hybrids were harvested and the F_2_ generation will be further investigated to analyze genetic regularity by re-sequenced pooled DNA of white color fruit and cloning mutations based on MutMap methods or map-based clone.

## 5. Conclusions

Abundant SNPs and indels were detected in the four EMS-induced eggplant mutants. The most common mutation type was C/G to T/A transitions. Also, the KEGG pathway and QPCR result suggest that Sme2.5_06210.1_g00004.1 may play a key role in eggplant anthocyanin synthesis.

## Figures and Tables

**Table 1:** Phenotypic description of wild typend four ethyl methanesulfonate induced eggplant mutants.

Line	Fruit Shape	Fruit Color	Sepal Color	Apex	Fruit Diameter	Fruit Length	Florescence
WT	Long	Aubergine	Purple	Concave	5 cm	30 cm	
J46-2	Long	Aubergine	Purple	Raised	5 cm	30 cm	
48-5	Oval	White	Green	Concave	10 cm	25 cm	
L6-5	Long	White	Green	Concave	5 cm	30 cm	
S26-1	Long	Purple black	Purple	Concave	5 cm	30 cm	Early

**Table 2:** Whole-genome resequencing of the wild type and four ethyl methanesulfonate-induced eggplant mutants.

Items	WT	J46-2	48-5	L6-5	S26-1
Clean base (bp)	56619013585	31640172900	30425850600	33955170600	33049549200
Average depth	58.2×	30.98×	29.98×	33.85×	31.89×
Genome Coverage	94.52%	98.70%	98.70%	98.67%	98.68%

**Table 3:** Genome-wide analysis of the SNPs (Single Nucleotide Polymorphisms) and indels’ numbers between the wild type and four ethyl methanesulfonate-induced eggplant mutants.

Item	Line	Variat_Number	Average (bp)	Max (bp)	Min (bp)
SNP	J46-2	294,966	2690	477,958	1
	48-5	331,414	2394	546892	1
	L6-5	477,587	1661	562,258	1
	S26-1	448,118	1770	568,513	1
Indel	J46-2	54,109	14,665	515,567	1
	48-5	61,014	13,005	538,563	1
	L6-5	75,273	10,542	550,336	1
	S26-1	75,500	10,510	547,033	1

**Table 4:** The number of the mutations on gene function in the four ethyl methanesulfonate -induced eggplant mutants.

Intem		J46-2	48-5	L6-5	S26-1
High-impact	nonsense mutations	18,638	20,737	28,489	27,220
	frameshift mutations	2323	2371	2285	2288
Modifier-impact	Intron mutation	96,836	108,394	159,089	147,403
	intergenic mutations	15,360	167,494	232,069	220,473
Low-impact	synonymous mutations	8767	10,276	13,236	13,071

**Table 5:** The high-impact mutation gene involved in the flavone and flavonol biosynthesis pathway.

Mutatant Line	Gene ID	Mutation	Mutation Type	Description
	Sme2.5_00384.1_g00003.1	nonsynonymous	G-T	F3’H, VvF3’h4; flavonoid 3′ hydroxylase
	Sme2.5_00928.1_g00014.1	nonsynonymous	C-T	flavonol-3-O-glycoside-7-O-glucosyltransferase 1
	Sme2.5_01087.1_g00007.1	nonsynonymous	A-G	F3H1; flavonoid 3′-hydroxylase
	Sme2.5_03930.1_g00006.1	nonsynonymous	G-C	flavonol-3-O-glycoside-7-O-glucosyltransferase 1
	Sme2.5_05662.1_g00003.1	nonsynonymous	C-T	flavonol-3-O-glycoside-7-O-glucosyltransferase 1
48-5	Sme2.5_13126.1_g00003.1	nonsynonymous	G-A	flavonol-3-O-glycoside-7-O-glucosyltransferase 1
	Sme2.5_13996.1_g00001.1	nonsynonymous	T-G	Flavonoid 3′ 5′-hydroxylase
	Sme2.5_24902.1_g00001.1	nonsynonymous	C-T	Flavonoid 3′,5′-hydroxylase 2-like
	Sme2.5_26073.1_g00001.1	nonsynonymous	T-C	COMT1; caffeic acid 3-O-methyltransferase
	Sme2.5_03583.1_g00011.1	nonsynonymous	T-C	anthocyanin 5-O-glucosyltransferase
	Sme2.5_00081.1_g00002.1	frameshift_deletion	GC-C	COMT2; caffeic acid 3-O-methyltransferase
	Sme2.5_01098.1_g00002.1	nonsynonymous	G-A	Anthocyanidin 3-O-glucosyltransferase 5-like
	Sme2.5_01191.1_g00004.1	nonsynonymous	T-G	Flavonoid 3′ 5′-hydroxylase
	Sme2.5_02030.1_g00005.1	nonsynonymous	A-G	Flavonol 3-O-methyltransferase
	Sme2.5_02274.1_g00006.1	nonsynonymous	T-C	flavonol-3-O-glycoside-7-O-glucosyltransferase 1
L6-5	Sme2.5_06210.1_g00004.1	nonsynonymous	C-T	flavonol-3-O-glycoside-7-O-glucosyltransferase 1
	Sme2.5_07919.1_g00001.1	nonsynonymous	G-A	UDP-glucosyl transferase family protein
	Sme2.5_12449.1_g00001.1	nonsynonymous	G-A	flavonoid 3′ hydroxylase
	Sme2.5_13126.1_g00003.1	nonsynonymous	G-A	flavonol-3-O-glycoside-7-O-glucosyltransferase 1
	Sme2.5_24611.1_g00001.1	nonsynonymous	G-A	anthocyanidin 3-O-glucosyltransferase 5-like
	Sme2.5_26073.1_g00001.1	nonsynonymous	T-C	COMT1; caffeic acid 3-O-methyltransferase
	Sme2.5_00081.1_g00002.1	frameshift_deletion	GC-C	COMT2; caffeic acid 3-O-methyltransferase

## Supplementary Materials

The following are available online at https://www.mdpi.com/2073-4425/10/8/595/s1, Table S1: The primers used for q-PCR.; Table S2: Pathway assignment based on high-impact mutation gene in the four EMS-induced mutants.; Table S3: The gene IDs involved in the anthocyanin synthesis. Figure S1: The nucleotide and protein sequence alignment of Sme2.5_06210.1_g00004.1 and Sme2.5_00228.1_g00013.1; Figure S2: The Sme2.5_06210.1_g00004.1 expression in four natural eggplant population.
